# Reproductive and endocrine responses to short-term safflower seed supplementation in anestrous ewes synchronized with progestagen

**DOI:** 10.5194/aab-69-421-2026

**Published:** 2026-08-17

**Authors:** Metehan Kutlu, Halef Doğan

**Affiliations:** 1 Department of Obstetrics and Gynaecology, Faculty of Veterinary Medicine, Necmettin Erbakan University, Konya, 42310, Türkiye; 2 Department of Obstetrics and Gynaecology, Faculty of Veterinary Medicine, Tekirdag Namik Kemal University, Tekirdag, 59030, Türkiye

## Abstract

This study investigated the effects of short-term supplementation with safflower seed, a linoleic-acid-rich oilseed, on reproductive performance and endocrine responses in Hungarian Merino ewes during the non-breeding season. Forty-four clinically healthy multiparous ewes were allocated to two equal groups: a control group and a safflower group. Ewes in the safflower group ( n=22 ) received 75 g ewe^−1^ d^−1^ of whole safflower seed in addition to the standard ration during the 7 d sponge period, whereas control ewes ( n=22 ) received no oilseed supplementation. All ewes were treated with an intravaginal sponge containing 60 mg medroxyprogesterone acetate for 7 d during April. At sponge removal, 500 IU equine chorionic gonadotropin (eCG) was administered intramuscularly. Ewes showing estrus were mated with proven fertile Merino rams (ewe : ram ratio of 5:1 ). Blood samples were collected on day 0, day 7, and immediately after mating on the day of estrus to determine serum estradiol (E2) and 13,14-dihydro-15-keto prostaglandin F2 α (PGFM) concentrations. Pregnancy was diagnosed by transabdominal ultrasonography 50 d after mating, and reproductive parameters were calculated. Under the present experimental conditions, short-term safflower seed supplementation did not produce statistically detectable improvements in estrus rate, pregnancy rate, lambing rate, or litter size compared with the control group ( p>0.05 ). Serum E2 concentrations increased, whereas PGFM concentrations decreased over time in both groups; however, no treatment effect or treatment × time interaction was detected. In conclusion, supplementation with 75 g ewe^−1^ d^−1^ of whole safflower seed during progestagen treatment was not associated with measurable improvements in reproductive outcomes or detectable changes in circulating E2 and PGFM concentrations.

## 1 Introduction

Rural sheep farmers experience considerable economic difficulties stemming from multiple challenges, including harsh environmental conditions, limited grazing areas, nutritional deficiencies, and prolonged postpartum anestrus periods (Nel et al., 2023; Ngcobo et al., 2026). In an economically sustainable sheep production system, two lambings per year can be achieved through effective reproductive control. However, to enhance reproductive parameters, it is essential to provide the ewes with an adequate and high-quality nutritional regimen (Aouina et al., 2025). Livestock intensively farmed under modern production systems typically have limited or no access to fresh pasture. Moreover, preserved forage contains low concentrations of polyunsaturated fatty acids; therefore, animal diets are frequently supplemented with fats derived from oilseeds rich in linoleic acid (Wathes et al., 2007).

Nutritional strategies, including dietary lipid supplementation, have been widely investigated as practical approaches to improve reproductive performance in ruminants (Yadav et al., 2019). Lipid supplementation may influence reproductive function through several mechanisms involving the hypothalamus, anterior pituitary, ovary, and uterus (Rahbar et al., 2014). Polyunsaturated fatty acids (PUFAs), especially n-6 and n-3 fatty acids, are essential nutrients because animals cannot synthesize them de novo and must obtain them from the diet (Wathes et al., 2007). Among these, linoleic acid is an essential n-6 PUFA that may be desaturated and elongated to arachidonic acid after escaping ruminal biohydrogenation and being absorbed from the digestive tract. Arachidonic acid is the main precursor for prostaglandin F2 α synthesis and may therefore influence uterine prostaglandin secretion and circulating concentrations of 13,14-dihydro-15-keto prostaglandin F2 α (PGFM), the major metabolite of prostaglandin F2 α (PGF2 α ) (Funston, 2004; Weems et al., 2006).

The possible reproductive effects of lipid supplementation are not limited to prostaglandin metabolism. Dietary fat may also affect ovarian steroidogenesis by increasing the availability of cholesterol, which is the primary substrate for steroid hormone synthesis, including estradiol (Grummer and Carroll, 1991; Bao et al., 1995). Therefore, oilseed supplementation rich in unsaturated fatty acids may potentially alter endocrine responses related to follicular activity, estradiol secretion, luteal function, and uterine prostaglandin production. Previous studies have reported that dietary sources rich in n-6 fatty acids may influence prostaglandin secretion and PGFM concentrations in ruminants (Petit et al., 2004; Grant et al., 2005; Silvestre et al., 2011). However, the magnitude of these effects may depend on species, physiological status, lipid source, dose, duration of supplementation, and the use of hormonal synchronization protocols.

Safflower (*Carthamus tinctorius* L.) is an ancient oilseed plant belonging to the Compositae family and has become increasingly important as a drought-resistant crop, particularly under conditions associated with climate change (Gümüş and Küçükersan, 2016). Safflower seeds contain approximately 20 %–40 % oil, and oleic, linoleic, stearic, and palmitic acids constitute 96 %–99 % of safflower oil (Coşge et al., 2007; Knowles, 1982; Sabzalian et al., 2008). Depending on the variety, the proportions of oleic and linoleic acids may vary between 10 %–32 % and 58 %–81 %, respectively (Coşge et al., 2007; Sabzalian et al., 2008). Because of its high linoleic acid content, safflower seed may represent a practical dietary source of n-6 PUFA for ruminants. In this context, safflower supplementation may have the potential to influence prostaglandin metabolism through the linoleic acid–arachidonic acid–PGF2 α pathway and steroid hormone synthesis through lipid-related changes in ovarian function.

In addition to its fatty acid profile, safflower contains several polyphenolic compounds, including lignans, flavones, and serotonin derivatives. Lignans and flavones are known to possess phytoestrogenic activity and may interact with estrogen receptors (Hong et al., 2002; Cho et al., 2011). Therefore, these compounds may influence estrogen-dependent reproductive processes, including follicular activity, estrus behavior, reproductive tract function, and circulating estradiol concentrations. Safflower-derived compounds have previously been associated with estrogen-related biological effects, particularly in models of estrogen deficiency and bone metabolism (Cho et al., 2011; Yuk et al., 2002). Moreover, safflower has traditionally been used for menstrual disorders and female infertility in some traditional medicine practices (Yang et al., 1993; Dajue and Mündel, 1996). Although most of these effects have been reported in non-ruminant or biomedical models, they provide a biological basis for investigating whether safflower supplementation can influence estradiol concentrations and reproductive outcomes in synchronized ewes.

Despite the potential effects of safflower seed on prostaglandin metabolism, steroidogenesis, and estrogen-related reproductive processes, there is limited information on its use as a nutritional supplement for improving reproductive performance in ewes. In particular, the effects of short-term whole safflower seed supplementation during progestagen-based estrus synchronization in anestrous ewes remain unclear. Because the non-breeding season is characterized by reduced reproductive activity, it is important to determine whether a short-term nutritional intervention during synchronization can support endocrine responses and reproductive outcomes.

Based on these mechanisms, we hypothesized that short-term dietary supplementation with safflower seed during progestagen-based synchronization may modulate circulating E2 and PGFM concentrations and thereby potentially influence reproductive outcomes. Therefore, the objective of the present study was to determine whether supplementation with whole safflower seed during the 7 d sponge period affects estrus response, pregnancy and lambing outcomes, and circulating E2 and PGFM concentrations in anestrous Hungarian Merino ewes synchronized with a short-term progestagen protocol.

## 2 Material and methods

### 2.1 Animals, location, diets, and experimental design

This study was conducted on a commercial sheep farm (lat: 37°26^′^22.04^′′^ N, long: 35°44^′^01.75^′′^ E, alt: 120 m) in Adana Province in Türkiye during the non-breeding season (April) in 2022. A total of 44 clinically healthy adult multiparous Hungarian Merino ewes, 3–4 years of age and weighing 55–60 kg, were used. The body condition score of the ewes ranged from 2.5 to 3.5. All ewes received antiparasitic treatment 1 month before the beginning of the experiment, and water was provided ad libitum throughout the study. Ewes were housed in a semi-open sheep barn throughout the study.

The cyclic status of the ewes was determined based on serum progesterone concentrations on day 0. Progesterone concentrations below 1 ng mL^−1^ were considered to be indicative of the absence of functional luteal activity, confirming that all ewes were in anestrus before sponge insertion.

In the study, 44 adult Hungarian Merino ewes were randomly allocated to treatment groups after balancing for body weight, parity, and body condition score. Ewes in the control group ( n=22 ) were not given any oilseeds. Ewes in the safflower group ( n=22 ) were fed 75 g ewe^−1^ d^−1^ of whole safflower seeds in addition to the standard ration during the 7 d sponge period.

Whole safflower seeds were used without any grinding or mechanical processing. During the supplementation period, 75 g ewe^−1^ d^−1^ of safflower seed was collectively offered to the safflower group by sprinkling the seeds onto the standard ration at feeding. The supplemented ration was completely consumed, and no refusal of safflower seed was observed throughout the experimental period.

Ewes were fed twice daily at 12 h intervals, for 2 h at 08:00 and 20:00, and water was provided ad libitum throughout the study. All ewes were fed a standard ration consisting of 53.5 % roughage and 46.5 % concentrate, starting 1 month prior to synchronization and continuing until intravaginal sponges were inserted. Chopped alfalfa hay and chopped wheat straw were used as roughage sources at proportions of 60 % and 40 %, respectively. Diets were formulated according to NRC (2007) using OptiTMR Pro 4.0.33. (Table 1).

In the present study, the amount of safflower seed was limited to avoid excessive fat intake that could adversely affect rumen fermentation. Considering the fact that the high-linoleate safflower seed used contained 31 % crude fat, supplementation at 75 g d^−1^ provided approximately 23.25 g d^−1^ of additional fat per ewe. This level helped keep the total dietary fat content below the recommended upper limit for ruminants, which is 6 %–7 % of dry matter. Therefore, 75 g d^−1^ of safflower seed was selected as a safe and controlled supplementation level to provide energy and unsaturated fatty acid support during the pre-mating period.

**Table 1:** Ingredients and chemical composition of the diets supplied to ewes.

	Control group	Safflower group
Ingredients (kg d^−1^ per head)		
Alfalfa hay	0.60	0.60
Wheat straw	0.40	0.40
Barley	0.85	0.85
Safflower	–	0.075
Salt	0.02	0.02
Vitamin-Mineral^1^	0.06	0.06
Chemical composition (% of DM)		
Crude protein	11.17	11.27
Fat	1.98	3.24
ADF	28.52	30.26
NDF	42.79	43.08
Ash	5.70	5.56
ME^2^ (Mcal kg^−1^)	2.38	2.37

^1^ Vitamin–mineral (kg): 15 000 000 IU vitamin A, 3 000 000 IU vitamin D3, 30 000 mg Vitamin E, 150 000 mg Niacin, 10 000 mg Cu, 800 mg I, 150 mg Co, 150 mg Se, 50 000 mg Mn, 50 000 mg Fe, 50 000 mg Zn, 6800 mg organic Mn, 1400 mg organic Cu, 6800 mg organic Zn, 6800 mg organic Fe, 50 mg organic Se. ^2^ Calculated using the OptiTMR Pro 4.0.33.

### 2.2 Feed analysis

Samples of the roughage and concentrate feeds offered during the trial were collected and evaluated for dry matter, crude protein, ether extract, and ash contents in accordance with AOAC (1990) procedures. Acid detergent fiber (ADF) and neutral detergent fiber (NDF) contents were determined using the procedure described by Van Soest et al. (1991). Fiber analyses were carried out with an ANKOM200 Fiber Analyzer (ANKOM Technology Corp., NY) using heat-stable α -amylase and sodium sulfite during the analysis.

Lipid extraction was conducted in duplicate following the method reported by Bligh and Dyer (1959). Fatty acid methyl esters were prepared from the extracted lipids according to Ichihara et al. (1996). Briefly, 4 mL of 2 M KOH and 2 mL of n -heptane were added to 25 mg of extracted oil. The mixture was vortexed for 2 min at room temperature and then centrifuged at 4000 rpm for 10 min. The upper-heptane phase was subsequently subjected to gas chromatographic analysis. The chemical composition of the safflower material used in the study is shown in Table 2.

**Table 2:** Chemical composition of safflower (*Carthamus tinctorius* L.) used in the study.

Item	Safflower
Nutrient composition (%)	
Dry Matter	93.69
Crude protein	12.73
Fat	31.06
ADF	46.82
NDF	67.03
Ash	2.25
Fatty acids (%)	
Palmitic acid (C16:0)	6.91
Palmitoleic acid (C16:1)	0.07
Stearic acid (C18:0)	2.80
Oleic acid (C18:1 n9 )	23.41
Vaccenic acid (C18:1 n7 )	1.49
Linoleic acid (C18:2 n6 )	61.70
Alpha linolenic acid (C18:3 n3 )	0.23

Chemical composition was analyzed by standard AOAC, Van Soest, and gas chromatographic methods.

### 2.3 Synchronization, estrus detection, and mating

All ewes were treated with a vaginal sponge containing progestagen (60 mg medroxyprogesterone acetate, Esponjavet^®^, Hipra, Spain) for 7 d during the non-breeding season (April) (day 0: insertion of vaginal sponge; day 7: removal of the vaginal sponge). At sponge removal, 500 IU eCG (Oviser^®^, Hipra, Spain) was administered intramuscularly. Estrus detection using teaser rams commenced 12 h after sponge removal. Observations were made in the morning (07:00–08:00) and evening (19:00–20:00). Ewes detected in estrus were hand mated with rams of proven fertility (ewe : ram ratio of 5:1 ).

The second service included ewes that either did not conceive after the first service or did not exhibit estrus during the first service period. Rams were kept with the flock after the first service, and ewes returning to or showing estrus approximately 17 d later were naturally mated by the same proven fertile Merino rams. Reproductive outcomes were therefore calculated separately for first service, second service, and overall services.

### 2.4 Blood collection and hormonal measurements

On days 0 and 7 and immediately after mating on the day of estrus, blood samples were collected from the jugular vein using non-heparinized vacuum tubes (9 mL). Serum was obtained by centrifugation at 3500 rpm for 15 min and stored at - 20 °C until analysis. Serum progesterone concentrations were measured using a sheep progesterone ELISA kit (catalog no. 201-07-0084, SunRed) with a sensitivity of 0.048 ng mL^−1^ for samples with progesterone concentrations between 0.05 and 15 ng mL^−1^. Serum estradiol (E2) levels were measured using a sheep estradiol ELISA kit (catalog no. 201-07-1030, SunRed) with a sensitivity of 0.925 pg mL^−1^ for samples between 1 and 300 pg mL^−1^. Serum 13,14-dihydro-15-keto prostaglandin F2 α (PGFM) was measured using a sheep PGFM ELISA kit (catalog no. 201-07-2144, SunRed) with a sensitivity of 2.848 pg mL^−1^ for samples between 3 and 900 pg mL^−1^. All assay procedures were performed according to manufacturer's instructions.

### 2.5 Ultrasonography and calculation of reproductive parameters

In all ewes, transabdominal ultrasound examination (Hitachi EUB-405, 3.5 MHz convex probe, Japan) was performed to diagnose pregnancy on day 50 post-mating. Pregnancy was confirmed by the detection of a gestational sac, fetal fluids, a fetus, or placentomes. Litter size was recorded at parturition. The reproductive parameters were calculated as follows (Kutlu and Akbulut, 2025):

- estrus rate (ER) = (the number of ewes showing estrus behaviors) / (the number of ewes receiving intravaginal sponge) × 100
- pregnancy rate (PR) = (the number of pregnant ewes) / (the number of ewes receiving intravaginal sponge) × 100
- lambing rate (LR) = (the number of lambing ewes) / (the number of pregnant ewes in each group) × 100
- litter size (LS) = (the number of total lambs) / (the number of lambing ewes in each group)

### 2.6 Statistical analysis

All statistical analyses and graphical illustrations were performed using the GraphPad Prism Version 8.0.2 (2019). The results were given as the percentage or mean and standard error (SEM). Reproductive parameters were compared between groups using the Chi-square test or Fisher's exact test, as appropriate. The normality of serum E2 and PGFM concentrations was assessed using the Shapiro–Wilk test. Serum E2 and PGFM concentrations were analyzed using a repeated-measures general linear model, including treatment group, sampling time, and treatment × sampling time interaction as fixed effects. When significant effects were detected, post hoc multiple comparisons were performed. Statistical significance was set at p < 0.05.

## 3 Results

**Figure 1:**
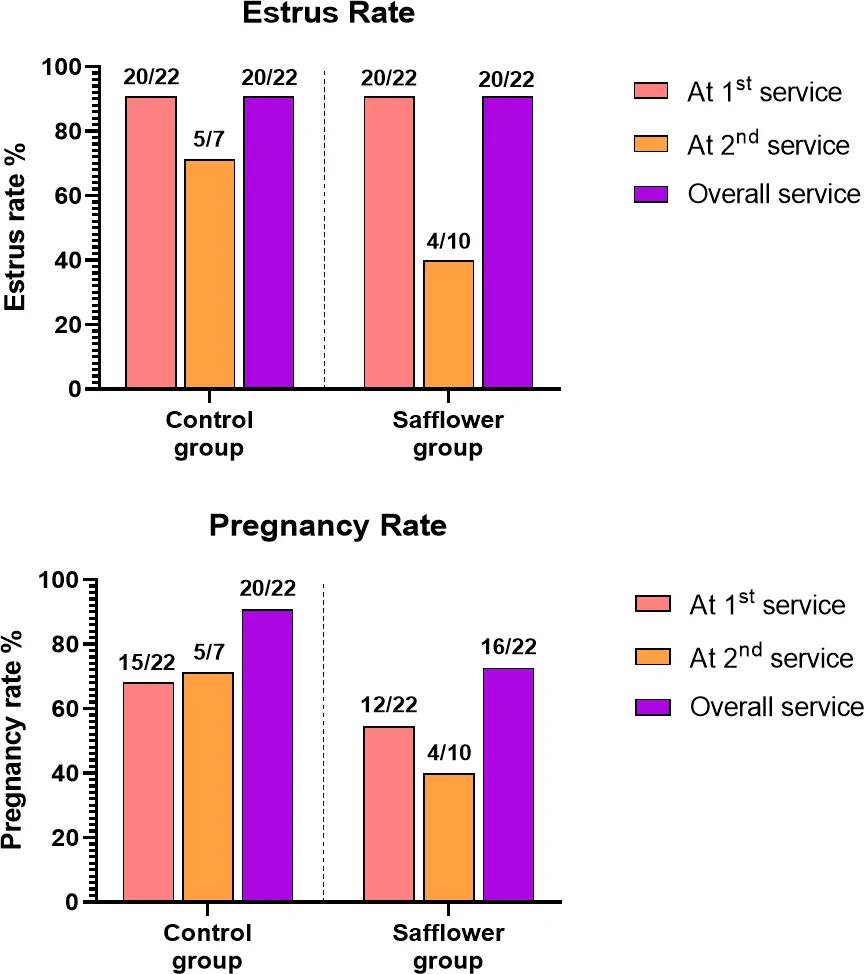
Estrus rate and pregnancy rate in study groups at the end of the study. There were no statistically significant differences between the control and safflower groups at the first, second, and overall service.

Estrus rate, pregnancy rate, lambing rate, number of lambs, and litter size were determined in the control and safflower groups at the first, second, and overall service. The reproductive parameters are presented in Fig. 1 and Table 3.

There were no statistically significant differences in any of the reproductive parameters at the first, second, and overall services between the groups ( p > 0.05).

**Figure 2:**
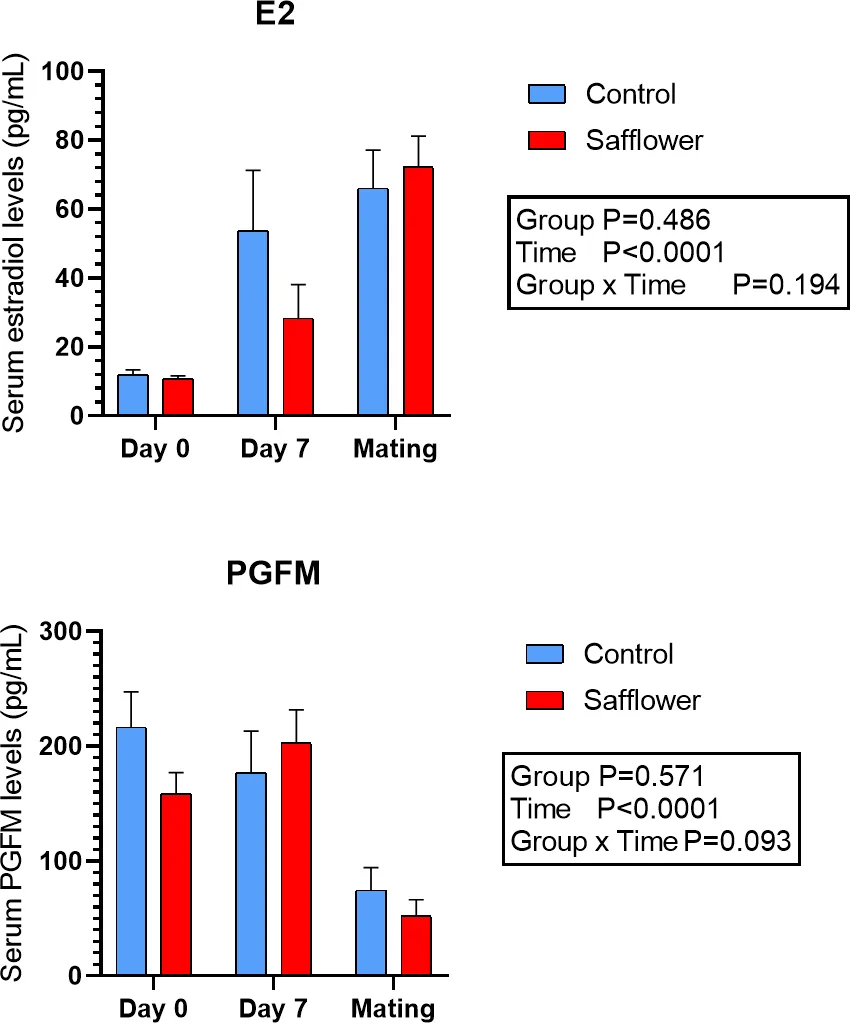
Changes in mean ± SEM serum estradiol (E2) and 13,14-dihydro-15-keto prostaglandin F2 α (PGFM) concentrations on day 0, day 7, and at mating in the control and safflower groups.

Serum E2 and PGFM concentrations measured on day 0, day 7, and the day of mating are presented in Table 4 and Fig. 2. For E2, there was a significant effect of time ( p < 0.0001), but no significant effect of treatment group ( p=0.489 ) or treatment group × time interaction ( p=0.194 ). For PGFM, there was a significant effect of time ( p < 0.0001), but no significant effect of treatment group ( p=0.571 ) or treatment group × time interaction ( p=0.093 ). Serum E2 concentrations increased from day 0 to the day of mating in both groups, whereas serum PGFM concentrations decreased markedly on the day of mating. Overall, no significant effect of safflower supplementation on serum E2 or PGFM concentrations was detected during the synchronization period.

**Table 3:** Reproductive parameters in control and safflower groups at the end of the study.

	At first service			At second service			Overall services		
Reproductive	Control	Safflower	p	Control	Safflower	p	Control	Safflower	p
parameters	group	group		group	group		group	group	
	( n=22 )	( n=22 )		( n=7 )^**^	( n=10 )^**^		( n=22 )	( n=22 )	
Estrus rate	90.9 % (20/22)	90.9 % (20/22)	> 0.999	71.40 % (5/7)	40 % (4/10)	0.201	90.9 % (20/22)	90.9 % (20/22)	> 0.999
Pregnancy rate	68.2 % (15/22)	54.5 % (12/22)	0.353	71.40 % (5/7)	40 % (4/10)	0.201	90.9 % (20/22)	72.7 % (16/22)	0.117
Lambing rate	100 % (15/15)	100 % (12/12)	> 0.999	100 % (5/5)	100 % (4/4)	> 0.999	100 % (20/20)	100 % (16/16)	> 0.999
Number of lambs	25	15		7	4		32	19	
Single	6	10		3	4		9	14	
Twin	8 (16)	1 (2)		2 (4)	–		10 (20)	1 (2)	
Triplets	1 (3)	1 (3)		–	–		1 (3)	1 (3)	
Litter size	1.67 (25/15)	1.25 (15/12)	0.144	1.4 (7/5)	1 (4/4)	0.713	1.6 (32/20)	1.19 (19/16)	0.5

Statistical analysis showed no significant difference between the groups ( p>0.05 ). For the second service, denominators represent ewes that either failed to conceive after the first service or did not exhibit estrus during the first service period.

**Table 4:** Serum estradiol (E2) and 13,14-dihydro-15-keto prostaglandin F2 α (PGFM) levels in the study.

	Control group ( n=22 )	Safflower group ( n=22 )	p
	(mean ± SEM)	(mean ± SEM)	
Serum E2 levels (pg mL^−1^)			
Day 0	12.04 ± 1.36 ^a^	10.71 ± 0.92 ^a^	G: 0.486
Day 7	53.73 ± 17.56 ^ab^	28.38 ± 9.71 ^a^	T: < 0.0001
Mating	66.04 ± 11.10 ^b^	72.25 ± 8.98 ^b^	G × T: 0.194
Serum PGFM levels (pg mL^−1^)			
Day 0	216.33 ± 30.99 ^a^	158.97 ± 18.25 ^a^	G: 0.571
Day 7	176.85 ± 36.37 ^a^	202.75 ± 28.80 ^a^	T: < 0.0001
Mating	74.67 ± 19.72 ^b^	52.14 ± 14.16 ^b^	G × T: 0.093

Day 0: insertion of intravaginal sponge; day 7: removal of vaginal sponge. G: group; T: time; G × T: interaction. ^a,b^ Different superscript letters within the same column indicate significant differences among sampling times ( p<0.05 ).

## 4 Discussion

Safflower (*Carthamus tinctorius* L.) is a drought-tolerant forage and oilseed whose seeds characteristically contain high proportions of polyunsaturated fatty acids (PUFAs) – most notably linoleic acid ( n-6 ) – together with polyphenolic constituents (Yaginuma et al., 2002; Landau et al., 2005; Peiretti, 2009). As a feed ingredient, safflower and its products have been evaluated under field conditions for their effects on growth, fetal development, neonatal survival, and milk composition (Bolte et al., 2002; Bouattour et al., 2006; Peiretti, 2009; Afshar et al., 2021; Bottger, 2001). Although palatability can be lower than that of other oilseeds, this drawback is commonly alleviated when safflower is incorporated into mixed rations (Smith, 1996; Sudhamayee et al., 2004). This study investigated whether short-term supplementation with whole safflower seeds, a linoleic-acid-rich oilseed, modulates reproductive performance and circulating hormonal dynamics in anestrous ewes synchronized with a progestagen protocol. Across first, second, and overall services, estrus, pregnancy, and lambing rates were comparable between the safflower-supplemented and control groups, and no pregnancy loss was observed between pregnancy diagnosis and lambing (Table 3). Regarding endocrine responses, serum E2 showed a significant time effect over the synchronization period but no group effect, while the prostaglandin F metabolite (PGFM) declined markedly toward mating in both groups, again, with no statistically significant between-group differences detected (Table 4). A slight, non-significant increase in PGFM after 7 d of safflower feeding was noted but was not associated with measurable improvements in fertility outcomes.

The lack of improvement in reproductive indices in our anestrous ewes appears to differ from some previous reports in cattle and goats in which lipid supplementation, particularly n-6 -rich sources, has been linked to enhanced luteal function, shifts in eicosanoid profiles, and higher conception rates (Grummer and Carroll, 1991; El Banna et al., 2005; Grant et al., 2005; Ravikiran et al., 2022). For instance, diets high in n-6 fatty acids may favor series-2 prostaglandin production (Petit et al., 2004), and safflower oil supplementation during the transition period increased serum PGFM in dairy cows (Silvestre et al., 2011). In ewes, pre-mating n-6 supplementation has likewise been associated with higher plasma progesterone and PGFM (Soydan et al., 2020). Species-level differences, physiological state (postpartum versus anestrus), supplementation duration, and the physical form and dose of the supplemental lipids may have contributed to these divergent outcomes.

Dietary linoleic acid that escapes ruminal biohydrogenation can be elongated and desaturated to arachidonic acid – the substrate for PGF2 α synthesis – thereby potentially influencing luteolysis, ovulation, and uterine function (Funston, 2004; Weems et al., 2006). Fat supplementation can also elevate circulating cholesterol, a precursor for steroidogenesis, and modulate lipoprotein delivery to ovarian cells (Grummer and Carroll, 1991; Bao et al., 1995). In the present study, however, the supplementation protocol (75 g ewe^−1^ d^−1^ of whole safflower seed for 7 d) may not have provided sufficient exposure to linoleic acid to induce measurable changes in systemic lipid metabolism, steroidogenic activity, or uterine eicosanoid production within the hormonal milieu of the progestagen-based synchronization protocol.

The endocrine responses observed in the present study are more likely to reflect protocol-driven physiological changes rather than an effect of safflower supplementation. Serum E2 concentrations increased toward mating in both groups, most likely reflecting follicular development stimulated after sponge removal and eCG administration. However, this increase was not influenced by safflower supplementation as no significant treatment effect or treatment × time interaction was detected. Conversely, serum PGFM concentrations declined markedly on the day of mating in both groups, suggesting that PGFM dynamics were primarily time-dependent and were not significantly modified by safflower seed supplementation. The absence of detectable endocrine and reproductive responses may also be related to the relatively short supplementation period. In addition, the use of whole safflower seed may have limited the intestinal availability of linoleic acid because a substantial proportion of dietary unsaturated fatty acids undergoes ruminal biohydrogenation before absorption. Consequently, the amount of biologically available n-6 polyunsaturated fatty acids reaching the small intestine may have been insufficient to induce measurable endocrine or reproductive responses during the synchronization period.

Several design features likely limited the detectable effect size: (1) the supplementation (7 d during sponge insertion) was short compared with studies reporting endocrine or fertility benefits from lipid supplementation; (2) the use of whole seeds rather than rumen-protected forms likely increased ruminal PUFA hydrogenation and reduced post-ruminal delivery; (3) the safflower used contained linoleic acid at the lower end of reference ranges, potentially attenuating the expected n-6 effects; (4) the strong exogenous control of a progestagen–eCG synchronization regimen may have overshadowed nutritionally mediated changes; and (5) although the groups were balanced ( n=22 per group), the present sample size may have limited the ability to detect moderate treatment effects. Because reproductive responses are influenced by multiple nutritional and endocrine factors, a short-term increase in dietary linoleic acid may not be sufficient to overcome the physiological constraints associated with seasonal anestrus, particularly when combined with a hormonally controlled synchronization protocol.

Within the constraints tested here, supplementation with 75 g ewe^−1^ d^−1^ of whole safflower seed for 7 d during progestagen synchronization in anestrous ewes was not associated with measurable increases in estrus expression, conception, or lambing. To better delineate safflower's potential in small-ruminant reproduction, future trials should extend supplementation to 3–6 weeks prior to and through synchronization with dose–response evaluation, compare whole seeds with rumen-protected lipid sources and high-linoleic with high-oleic cultivars, incorporate mechanistic endpoints (lipid profiles, mid-luteal progesterone, serial PGFM, LH pulses, luteal blood flow), stratify by physiological state, and increase sample size based on predefined minimal clinically important differences.

## 5 Conclusions

Short-term supplementation with 75 g ewe^−1^ d^−1^ of whole safflower seed during a 7 d progestagen-based synchronization protocol did not result in statistically detectable improvements in estrus response, pregnancy rate, lambing outcome, litter size, or circulating E2 and PGFM concentrations in anestrous Hungarian Merino ewes under the present experimental conditions. These findings suggest that the supplementation protocol evaluated in this study did not provide measurable reproductive or endocrine benefits. However, further studies using longer supplementation periods, rumen-protected lipid sources, dose–response designs, and larger sample sizes are needed to better define the potential role of safflower supplementation in improving reproductive performance in ewes.

## Data Availability

The datasets used and analyzed during the current study are available from the corresponding author on reasonable request.

## Code availability

The statistical code used in this study is available from the corresponding author upon reasonable request.
